# Postmortem Evaluation of Left Flank Laparoscopic Access in an Adult Female Giraffe (*Giraffa camelopardalis*)

**DOI:** 10.4061/2010/789465

**Published:** 2010-03-30

**Authors:** R. Pizzi, J. Cracknell, L. Dalrymple

**Affiliations:** ^1^Royal Zoological Society of Scotland, Edinburgh Zoo, 134 Corstorphine Road, EH12 6TS Edinburgh, UK; ^2^Zoological Medicine Ltd, 79A Garvock Hill, Dunfermline, KY12 7UT Fife, UK; ^3^Inglis Veterinary Centre, 120 Halbeath Road, Dunfermline, KY11 4LA Fife, UK; ^4^Marwell Wildlife, Colden Common, Winchester, SO21 1JH Hampshire, UK

## Abstract

There are still few reports of laparoscopy in megavertebrates. The giraffe (*Giraffa camelopardalis*) is the tallest land mammal, and the largest ruminant species. An 18-year-old multiparous female hybrid giraffe, weighing 650 kg, was euthanized for chronic health problems, and left flank laparoscopy was performed less than 30 minutes after death. Safe primary access was achieved under visualisation using an optical bladed trocar (Visiport Plus, Tyco healthcare UK Ltd) without prior abdominal insufflation. A left paralumbar fossa approach allowed access to the spleen, rumen, left kidney, and intestines, but did not allow access to the reproductive tract which in nongravid females is intrapelvic in nature.

## 1. Case Report

Laparoscopy holds advantages over open surgery in zoo animals. Aside from smaller wounds, there is magnified visualisation of target organs [1]. Decreased postoperative pain, lower morbidity, lower rates of postoperative infection, and decreased risk of dehiscence are reported in humans and domestic animals [2, 3]. There are few reports of laparoscopy in megavertebrates, such as African elephants (*Loxodonta africana*) [4], white rhinoceros (*Ceratotherium simum*) [5], black rhinoceros (*Diceros bicornis*) [6], and Northern elephant seal (*Mirounga angustirostris*) [7]. There are also few published reports of surgery in giraffes [8–10], and the authors are unaware of any published reports of laparoscopy in giraffes. 

The giraffe (*Giraffa camelopardalis*) is the tallest land mammal and the largest ruminant [11, 12]. It is regarded as “Least Concern” from a conservation perspective by the International Union for the Conservation of Nature (IUCN), with total wild populations estimated between 110,000 and 150,000, but the West African subspecies (*G. C. peralta*) has been classified as endangered [13]. Differing numbers of subspecies have been proposed [14–18], and recent genetic work has even suggested that some subspecies may be distinct species [19]. 

A female, 16-year-old Rothschild's giraffe (*Giraffa camelopardalis rothschildi*) presented with chronic arthritis. This had been adequately managed for three years with phenylbutazone (Equipalazone, Powder, Arnolds, 1 g sachet) 2 g bid. During the six months prior to euthanasia the giraffe had begun to lose weight despite increased rations and a good appetite. Biochemistry and haematological profiles were within normal limits throughout the course of the weight loss. In the last few weeks prior to euthanasia the lameness increased and it was decided to euthanize the giraffe on welfare grounds. Food but not water was withheld for 36 hours before, as for laparoscopy in cattle to reduce rumen fill and increase the potential operating space [20, 21], and laparoscopy was performed less than 30 minutes after euthanasia.

The preanaesthesia, live weight was 673 kg. Anaesthesia was induced using a premedication of haloperidol (Haldol Injection, Janssen-Cilag, 5 mg/mL) 40 mg intramuscularly (IM), then 60 minutes later ketamine (Narketan 10, Vetoquinol, 100 mg/mL) 1.3 g and medetomidine (Zalopine, Orion Pharmaceuticals, 10 mg/mL) 35 mg IM. Euthanasia was achieved with cinchocaine HCl 1.25 g and secobarbital sodium 20 g IV (Somulose, Arnolds, 25 mg/mL and 400 mg/mL, respectively) via jugular cannulation. Anaesthesia was smooth, however, the haloperidol dose was four times that normally given for induction and led to difficulty in moving the animal into the induction stall due to the heavy sedation.

**Figure 1:**
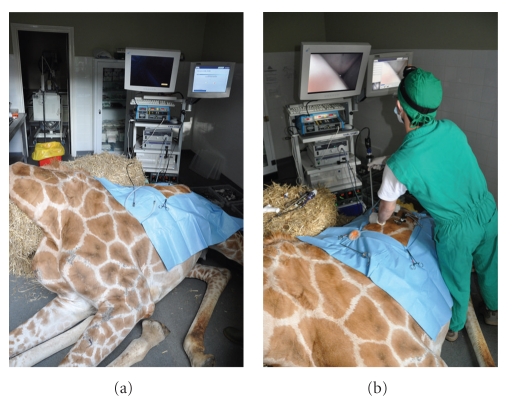
Positioning of the giraffe postmortem and the laparoscopy stack for the left flank laparoscopic approach.

**Figure 2:**
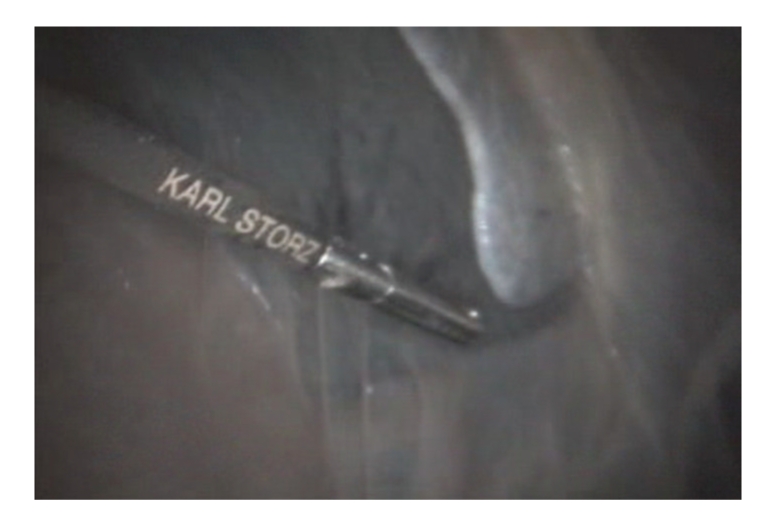
Laparoscopic examination of the caudal surface of the spleen and its attachment to the abdominal wall.

**Figure 3:**
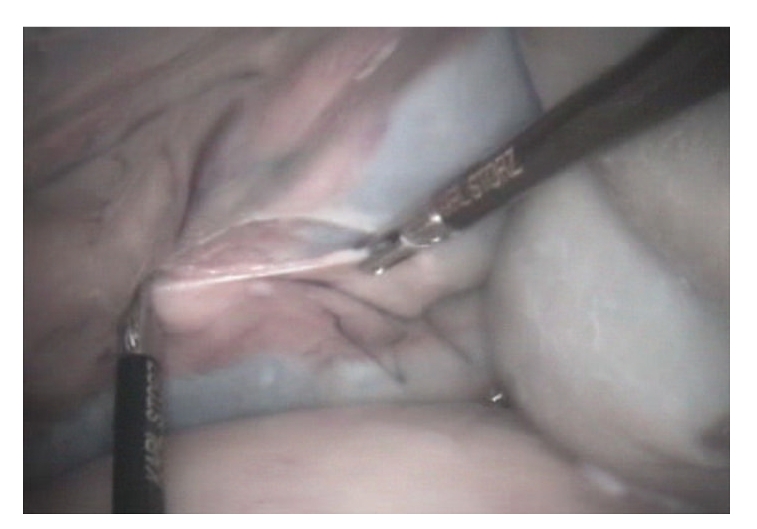
Dissection of the peritoneum to access the left kidney using 43 cm length 5 mm diameter bariatric instruments.

After euthanasia the giraffe was positioned in right lateral slightly oblique recumbency as it would be practical under anaesthesia. A 2 cm skin incision was made in the dorsal mid left paralumbar fossa. Access was achieved by using a cutting optical trocar (Visiport Plus 5–11 mm; Tyco healthcare UK Ltd), without prior insufflation, with visualisation provided by a 30 cm length 0 degree 10 mm diameter laparoscope (Karl Storz Endoscopy UK Ltd, Slough). Activation of the trigger fired and retracted a 1mm depth cutting blade, allowing controlled insertion of the cannula under visualisation through the abdominal muscles (Figure 1). Once the peritoneum was reached the cannula was gently advanced through this bluntly under visualisation. Laparoscopy was performed using a 58 cm length 10 mm diameter 0 degree human bariatric laparoscope (Karl Storz Endoscopy UK Ltd, Slough). Insufflation with carbon dioxide was performed at 12 mmHg intraabdominal pressure. A 150-watt Xenon light source provided sufficient illumination, with visualisation via a dedicated camera, processor, and software (Aida Compact, Karl Storz Endoscopy UK Ltd). Organ manipulation was performed with 43 cm length 5 mm diameter bariatric laparoscopic instruments. Laparoscopy at this site only allowed access to the caudal aspect of the spleen (Figure 2); dorsal and caudal walls of the rumen, lateral left kidney (Figure 3), portions of the ileum and colon, and the peritoneum. It was not possible to access the liver, right kidney, inguinal canals, uterus, or ovaries from this position. It was also not possible to visualise the left ureter, renal vessels, or adrenal gland. 

**Figure 4:**
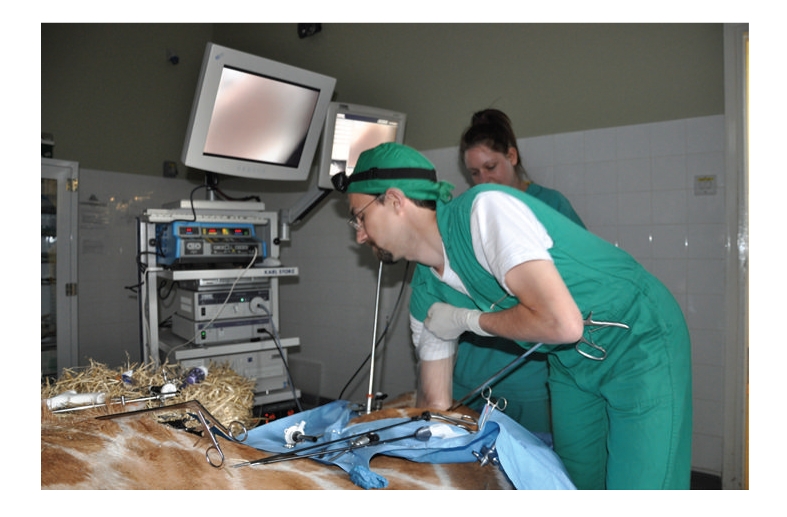
After laparoscopic exploration using standard 43 cm length bariatric laparoscopic instruments, a port site was enlarged to allow hand and arm access in a full length glove, while still maintaining a pneumoperitoneum.

As in other large ruminants, the operating space even with insufflation was extremely limited, with difficulty in organ retraction and limited visibility, due to the large rumen and voluminous intestinal tract. One port site was enlarged to allow the insertion of an arm in an arm-length glove, and the other port to allow the entry of a 95 cm length 12 mm width oesophageal foreign body forceps (Veterinary Instrumentation, Sheffield), but these resulted in no better visualisation or organ assess than with the orthodox 5 mm 43 cm bariatric instruments (Figure 4).

Thoracoscopy access was performed at numerous dorsal intercostals and costochondral junction sites with a 30 cm length 10 mm diameter 30 degree laparoscope (Karl Storz Endoscopy UK Ltd, Slough), but again visualisation and access were severely limited by the wide, closely spaced ribs that did not allow much endoscope angulation, and the relatively rigid fibrous lungs made atraumatic manipulation impossible.

**Figure 5:**
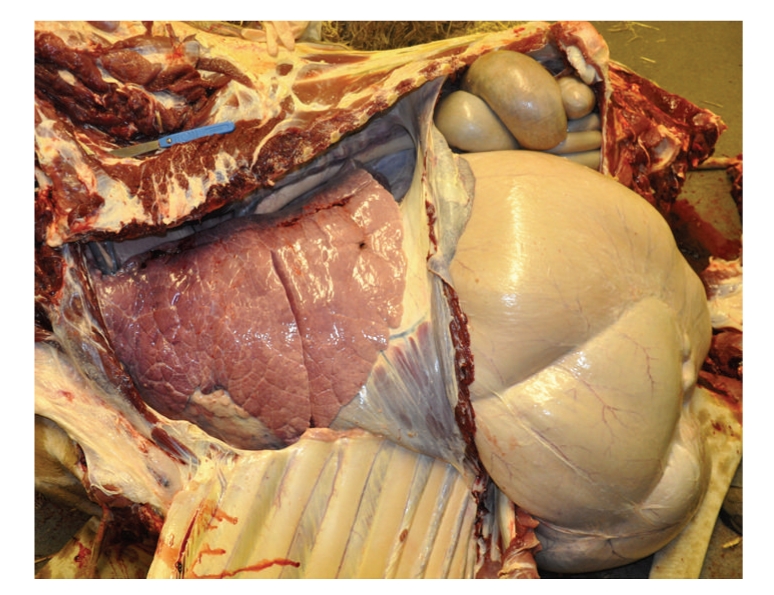
The initial postmortem examination approach consisted of removing the overlying abdominal and thoracic walls to validate the organ positions seen on laparoscopy. Note the wide flat ribs that may be thoracoscopy difficult by severely limiting the ability to manipulate the endoscope.

A full postmortem examination immediately following laparoscopy demonstrated no gross anatomic abnormalities and allowed proofing of the anatomic placements of the organs seen (Figure 5). No notable macroscopic pathology was evident aside from severe osteoarthritis lesions present in numerous limb joints, mild endocardiosis of the mitral valve, abomasal ulceration, likely due to chronic phenylbutazone use, and a multifocal bacterial dermatitis. The uterus and ovaries were confirmed to be entirely intrapelvic, despite the multiparous female having a recently weaned calf; consistent with findings in other adult females examined postmortem (Cracknell, unpublished data). The uterus extended less than 35 cm from the vulva. In cattle there is access to the female reproductive tract via a left flank laparoscopic approach [21–23]. The small reproductive tract in nongravid female giraffes may lend itself to transvaginal laparoscopic access, which warrants further investigation. It was not possible to attempt all other possible laparoscopic surgical approaches on this occasion due to time constraints. The giraffe was euthanized in the evening after the zoological collection was closed to the public. Time was limited by the fact that the laparoscopy attempts, full postmortem examination, dismembering and disposal of the body, and cleanup on site had to be completed before the zoo was opened to the public the next morning. 

Ruminant anatomy and the practicalities of safe positioning under anaesthesia are limitations to giraffe laparoscopy. Although dorsal recumbency for laparoscopy in cattle has been described [24, 25], this position has been associated with adverse cardiopulmonary and haemodynamic effects [26–29]. In cattle laparoscopy may be performed standing under sedation [21] which helps create a working space with insufflation, but it would be difficult to safely achieve in an adult giraffe. 

Optical trocars are utilised in human surgery for difficult primary access such as scarred, previously operated abdomens, or morbid obesity [30–32], and their use as been reported in equines [33]. While originally designed for use after blind insufflation by means of a verress needle, safe use without prior insufflation has been demonstrated in human surgery [32, 34, 35].

The limited organ visualisation and access possible from a left lateral laparoscopic approach limits its usefulness. Potential applications of a left lateral laparoscopic approach include a left abdominal cryptorchidectomy, biopsy of the left kidney, splenic biopsy or laparoscopic assisted intestinal biopsy. Thoracoscopy appears even more limited in application and would allow little more than a small endoscopic loop ligature lung biopsy to be performed. As negative or unsuccessful surgical procedures are rarely reported in the veterinary literature, it is hoped that this information may be helpful to veterinarians working in zoological collections, even if just to decide that laparoscopy may not be a viable option in a specific case.

The use of an optical cutting trocar appears to be a practical method of achieving primary laparoscopic access in the giraffe without the need for prior insufflation. While not suitable for all procedures, and despite few reports of surgery in giraffes in the literature [8–10], laparoscopy may add to the repertoire of potential diagnostic and surgical techniques available to the zoological surgeon when treating giraffes.

## References

[1] Lhermette P, Sorbel D (2008). An introduction to endoscopy and endosurgery. *BSAVA Manual of Endoscopy and Endosurgery*.

[2] Bailey JE, Pablo LS, Freeman LJ (1999). Anaesthetic and physiologic considerations fro veterinary endosurgery. *Veterinary Endosurgery*.

[3] Freeman LJ, Freeman LJ (1999). Introduction. *Veterinary Endosurgery*.

[4] Bokhout B, Nabuurs M, De Jong M (2005). Vasectomy of older bulls to manage elephant overpopulation in Africa: a proposal. *Pachyderm*.

[5] Radcliffe RW, Ferrell ST, Childs SE (2000). Butorphanol and azaperone as a safe alternative for repeated chemical restraint in captive white rhinoceros (*Ceratotherium simum*). *Journal of Zoo and Wildlife Medicine*.

[6] Portas TJ, Hermes R, Bryant BR, Goritz F, Thorne AR, Hildebrandt TB (2006). Anesthesia and use of a sling system to facilitate transvaginal laparoscopy in a black rhinoceros (*Diceros bicornis minor*). *Journal of Zoo and Wildlife Medicine*.

[7] Fauquier D, Gulland F, Haulena M, Spraker T (2003). Biliary adenocarcinoma in a stranded northern elephant seal (*Mirounga angustirostris*). *Journal of Wildlife Diseases*.

[8] Radcliffe RM, Turner TA, Radcliffe CH, Radcliffe RW (1999). Arthroscopic surgery in a reticulated giraffe (Giraffa camelopardalis reticulata). *Journal of Zoo and Wildlife Medicine*.

[9] Williams DC, Murison PJ, Hill CL (2007). Dystocia in a Rothschild giraffe leading to a caesarean section. *Journal of Veterinary Medicine A*.

[10] Davis MR, Langan JN, Mylniczenko ND, Benson K, Lamberski N, Ramer J (2009). Colonic obstruction in three captive reticulated giraffe (Giraffa camelopardalis reticulata). *Journal of Zoo and Wildlife Medicine*.

[11] Skinner JD, Smithers RHM (1990). *The Mammals of the Southern African Subregion*.

[12] Wilson DE, Treeder DM (2005). *Mammal Species of the World*.

[13] Fennessey J, Brown D (2009). Giraffa camelopardalis. *IUCN Red List of Threatened Species, Version 2009.1*.

[14] Ansell WF, Meester J, Setzer HW (1971). Familly giraffifdae. *The Mammals of Africa: An Identification Guide*.

[15] Dagg AI, Foster JB (1982). *The Giraffe: Its Biology, Behaviour, and Ecology*.

[16] Kingdon J (1997). *The Kingdon Field Guide to African Mammals*.

[17] East R (1998). *African Antelope Database*.

[18] Grubb PJ, Wilson DE, Reeder DM (2005). Artiodactyla. *Mammal Species of the World: A Taxonomic and Geographic Reference*.

[19] Brown DM, Brenneman RA, Koepfli K-P (2007). Extensive population genetic structure in the giraffe. *BMC Biology*.

[20] Trostle S, Freeman LJ (1999). Minimally invasive surgery of the large animal gastrointestinal system. *Veterinary Endosurgery*.

[21] Bleul U, Hollenstein K, Kahn W (2005). Laparoscopic ovariectomy in standing cows. *Animal Reproduction Science*.

[22] Wishart DF, Snowball JB (1973). Endoscopy in cattle: observation of the ovary in situ. *Veterinary Record*.

[23] Hendrickson DA, Freeman LJ (1999). Minimally invasive surgery of the reproductive system in large animals. *Veterinary Endosurgery*.

[24] Anderson DE, Gaughan EM, St-Jean G (1993). Normal laparoscopic anatomy of the bovine abdomen. *American Journal of Veterinary Research*.

[25] Mulon P-Y, Babkine M, Desrochers A (2006). Ventral laparoscopic abomasopexy in 18 cattle with displaced abomasum. *Veterinary Surgery*.

[26] Watney GCG (1986). Effects of posture and intraruminal pressure on the bronchial calibre of cattle during xylazine/halothane anaesthesia. *Research in Veterinary Science*.

[27] Klein L, Fisher N (1988). Cardiopulmonary effects of restraint in dorsal recumbency on awake cattle. *American Journal of Veterinary Research*.

[28] Wagner AE, Muir WW, Grospitch BJ (1990). Cardiopulmonary effects of position in conscious cattle. *American Journal of Veterinary Research*.

[29] Riebold T, Greene SA (2002). Ruminant anaesthesia. *Veterinary Anaesthesia and Pain Management Secrets*.

[30] Melzer A, Riek S, Roth K, Buess G (1995). Endoscopically controlled trocar and cannula insertion. *Endoscopic Surgery and Allied Technologies*.

[31] Cadeddu JA, Chan DY, Hedican SP (1999). Retroperitoneal access for transperitoneal laparoscopy in patients at high risk for intra-abdominal scarring. *Journal of Endourology*.

[32] Rabl C, Palazzo F, Aoki H, Campos GM (2008). Initial laparoscopic access using an optical trocar without pneumoperitoneum is safe and effective in the morbidly obese. *Surgical Innovation*.

[33] Desmaizières L-M, Martinot S, Lepage OM, Bareiss E, Cadoré J-L (2003). Complications associated with cannula insertion techniques used for laparoscopy in standing horses. *Veterinary Surgery*.

[34] Bernante P, Foletto M, Toniato A (2008). Creation of pneumoperitoneum using a bladed optical trocar in morbidly obese patients: technique and results. *Obesity Surgery*.

[35] Madan AK, Taddeucci RJ, Harper JL, Tichansky DS (2008). Initial trocar placement and abdominal insufflationin laparoscopic surgery. *Journal of Surgical Research*.

